# Reference Valence Effects of Affective S–R Compatibility: Are Visual and Auditory Results Consistent?

**DOI:** 10.1371/journal.pone.0095085

**Published:** 2014-04-17

**Authors:** Zhao Xiaojun, You Xuqun, Shi Changxiu, Gan Shuoqiu, Hu Chaoyi

**Affiliations:** 1 Shaanxi Key Laboratory of Behavior and Cognitive Neuroscience, Shaanxi Normal University, Xi'an, P. R. China; 2 School of Education, Anqing Normal College, Anqing, P. R. China; University of Leicester, United Kingdom

## Abstract

Humans may be faster to avoid negative words than to approach negative words, and faster to approach positive words than to avoid positive words. That is an example of affective stimulus–response (S–R) compatibility. The present study identified the reference valence effects of affective stimulus–response (S–R) compatibility when auditory stimulus materials are used. The researchers explored the reference valence effects of affective S–R compatibility using a mixed-design experiment based on visual words, visual pictures and audition. The study computed the average compatibility effect size. A *t*-test based on visual pictures showed that the compatibility effect size was significantly different from zero, *t* (22) = 2.43, *p*<.05 (M = 485 ms). Smaller compatibility effects existed when switching the presentation mode from visual stimuli to auditory stimuli. This study serves as an important reference for the auditory reference valence effects of affective S–R compatibility.

## Introduction

Some psychologists believe that the human reaction of evaluation is unconscious if not all of the encountered stimuli fall on a good or bad dimension. The human reaction of evaluation is presumed to have a transitory avoidance and approach behavioral tendency [1]–[3]. A recent study [3] identified the reference valence effects of affective stimulus–response (S–R) compatibility when visual stimulus materials were used (including visual text and visual images). Matching stimulus materials provides an explanation for the reference valence effects of affective stimulus–response (S–R) compatibility. The match between the stimulus and its referent is a key factor based on the valenced referent. The transitory approach behavioral tendency should be faster for the avoidance behavioral tendency when the target stimulus (such as a positive character) matches the referent (such as a positive personality trait). However, no studies have examined whether the matching account is a reasonable explanation for the reference valence effects of affective S–R compatibility when auditory stimulus materials are used.

### From the S–R compatibility effect to the affective S–R compatibility effect

Numerous accounts have interpreted the S–R compatibility effect [4]–[6]. In terms of reaction time and accuracy, the S-R compatibility effect showed that compatibility stimulus-response tasks were better than other tasks. Related research suggested that visual and response selections occur in different stages. In a simple present–absent detection task, participants were slower to respond ‘present’ when the single arrow pointed to the right (corresponding to the right hand). But, when the study changed the conditions (such as encouraging participants to process the identity of the arrow), the S–R compatibility effect was identified [7]. In addition, avoidance cues led to improved performance in cognitive control tasks [8]. A recent study found that an affective stimulus valence contributes to behavior that greatly leads to a compatible transformation in distance [9]. In spatial cognition, the absence of spatial compatibility effects when there is a strong temporal overlap suggests that response conflicts are caused by stimulus-related priming [10]. In the dual processing models of S–R compatibility, the processes of spatial and imitative compatibility are independent [11]. In cognitive mechanisms, negative compatibility effects are generated by perceptual mechanisms [12]. Similarly, reaction activation is affected by negative priming effects. This activation may be generated by a simplified image of a hand [13]. The latest research results cannot be interpreted with current accounts (specific-muscle-activation, evaluative response coding, distance-regulation, etc.) but are in agreement with the matching account [3]. The affective stimulus–response (S–R) compatibility effect demonstrates that participants are faster to avoid negative stimuli than to approach negative words. Similarly, participants are faster to approach positive stimuli than to avoid positive words [1]. In previous studies of visual positive and negative stimulation, it was not clear whether auditory stimuli have the same effect. The activity of mechanism is reflected in two aspects: valence effects and approach/avoidance action.

### Valence effects

Studies of valence effects are mainly concentrated in the following areas: gender differences, personality, emotions and brain cognition. The dependence of valence-specific effects in facial emotion perception on the perceiver's gender is unclear [14]. Regarding gender, small developmental increases occur more for the effects of valence than for arousal [15]. Regarding personality, positive adjectives are better recalled than negative adjectives when they are encoded in reference to the self [16]. Regarding trait identification, participants perform much better in positive versus negative behavior [17]. The valence of self-evaluative thoughts is thought to mediate the impact of personality traits on mood [18]. In the field of emotion, the effect of arousal on the connectivity within the emotional memory network depends on item valence [19] A valence-specific laterality effect is demonstrated in original stimuli when stimuli of the same emotion are presented as a block [20]. Experiments following ‘no think’ instructions for memories associated with emotionally negative material lead to significant memory suppression [21]. In addition, imagery manipulates emotional valence and arousal [22]. Mneimne found significant valence through visual field interactions [23]. In brain cognition, corrugator muscle activity are associated with left hemi-face dominance during high and low arousal negative picture blocks, whereas zygomaticus muscle activity are associated with right hemi-face dominance during high arousal positive picture blocks [24].

### Approach and avoidance action

Approach and avoidance motivation (or action) is very important for human function [3]. In a study with stimulation materials, an avoidance strategy was more effective in decreasing prejudice in a negative context rather than in a positive context [25]. Movement times are slower in the context of avoidance conflicts relative to approach conflicts [26]. In social cognition, some evidence indicates that avoidance temperament (neuroticism and negative affectivity) is a predictor of an approach goal, and approach temperament (extraversion and positive affectivity) is a predictor of an avoidance goal [27]. Approach/avoidance moderates the impact of comparison information on self-evaluation [28].

### Current study

Participants are faster to avoid negative stimuli than to approach negative words; similarly, participants are faster to approach positive stimuli than to avoid positive words [1]. The activity of mechanism is reflected in two aspects: valence effect, approach and avoidance action. Some studies on the valence effects of S–R compatibility exist, but studies on stimulation materials of audition are rare. The studies were based on vision, thus it is not clear if auditory stimuli have the same effect. One's perception of emotional content depends on auditory and visual cues. Auditory stimulus may affect reference valence effects of affective S–R compatibility. Auditory cues contain fundamental frequency changes during an utterance [29]–[30]. The related research shows that visual stimuli influence neural representation in the auditory cortex and may override auditory perceptions derived from auditory stimuli [31]. In our life, a lot of information belongs to auditory stimuli (e.g., the sound of the car). When crossing the street, it is assumed that the sound of speeding car can be heard, will you be more quickly to avoid this kind of negative stimuli? Visual and auditory stimulus are different for human to process according to the human physiology. In addition, the visual processing is a kind of image processing, while auditory processing is more a kind of meaning processing. Based on the above considerations, the present study aims to explore the auditory reference valence effects of affective S–R compatibility.

## Method

### Participants

Twenty-three college students between the ages of 18 and 20 (M = 19.0 years, SD = 0.71, 11 women and 12 men) participated in the experiment. All participants were Chinese speakers. All participants were right-handed and had normal or corrected-to-normal vision (auditory). All participants provided their written informed consent to participate in this study. The study was approved by the academic and ethics committee of school of education in Anqing Normal College. The academic and ethics committees approved this consent procedure. YUE FEI fought against the Jin race and became a national hero of the Chinese history. Before his departure to defend his country against the Jin army, his mother the tattooed four characters ‘jin zhong bao guo’ on his back to encourage him to serve his homeland with loyalty. QIN HUI is one of the ten biggest traitors in the history of China because he had YUE FEI executed for an ‘unwarranted’ count; an act which left a smell for ten thousand years. Thus, the reference objects for this experiment were YUE FEI and QIN HUI. All participants were familiar with the ancient Chinese characters (YUE FEI and QIN HUI) of which YUE FEI and QIN HUI were characteristic. All participants had clearly good and bad evaluations for YUE FEI and QIN HUI, respectively.

### Materials

A computer was used to present stimulus and record keyboard response. A 17-inch HPL1908w CRT computer monitor with 1440×900 resolution, true colour, and 60 Hz refresh rate was used. All experiment materials were presented through the DMDX stimulus presentation system. The sizes of the stimulus materials had default values. Distance to the screen was about 60 cm. The experiment material was Chinese material. There were 16 personality adjectives in the experiment. Each of the adjective was composed of 3–5 characters. The 16 personality adjectives were from factors of 16PF scale. Before the formal experiment, experimenter asked for informal participants to assess the adjectives in order to distinct them into positive and negative. In each group experiments, half of the adjective is positive (16), half of the adjective is negative (16).

Records of auditory experiment materials were produced by man using mandarin. Before formal experiment, the participants need to identify the contents of the tape recording and confirm that they can understand the recording materials. The auditory experiment materials embedded in the DMDX procedure. Auditory materials (taking up 1500 ms time) were played through mini sound box, which standed at the central position in front of computer. The experiment was conducted with the participants inside a moderately bright laboratory. The screen background was white, while visual stimulation was black (fixation point is black ‘+’).

### Design

The conducted study was a 2 (gender: male vs. female) × 3 (presentation mode: visual words vs. visual pictures vs. auditory stimuli) × 2 (reference objects: YUE FEI vs. QIN HUI) × 2 (valence of stimulate: positive vs. negative) × 2 (direction of movement: approach vs. avoidance) mixed design experiment. Gender was a between-group variable; the rest were within-group variables. The dependent variable was RT. Because all of the factors in the analysis except gender were within-subject manipulations, the design was adequately powered to detect medium-to-small effect sizes.

### Procedure

**Figure 1:**
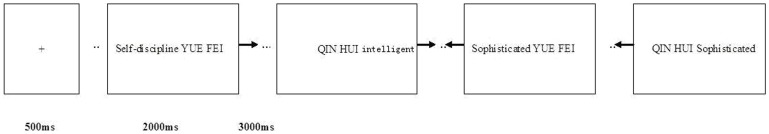
Positive approach - negative avoidance (consistent pattern). The Group 1 condition tested a consistent pattern based on visual words. A reference name appeared on the center of the screen, and a corresponding personality adjective appeared on either the left or the right side of the reference name.

**Figure 2:**
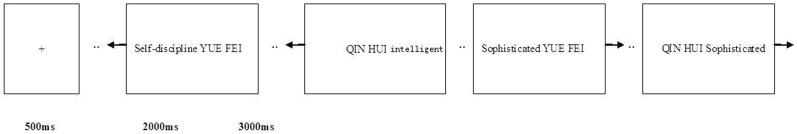
Positive avoidance – negative approach (inconsistent pattern). The Group 2 condition tested an inconsistent pattern based on visual words. A reference name appeared on the center of the screen, and a corresponding personality adjective appeared on either the left or the right side of the reference name.

**Figure 3:**
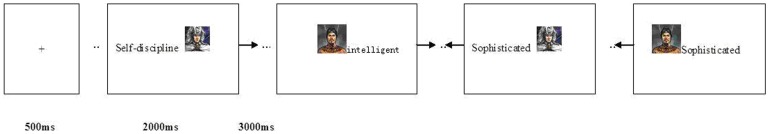
Positive approach - negative avoidance (consistent pattern, visual pictures). The Group 3 condition tested a consistent pattern based on visual pictures. A picture of the reference object was displayed on the screen, and a corresponding personality adjective appeared on either the left or the right side of the picture.

**Figure 4:**
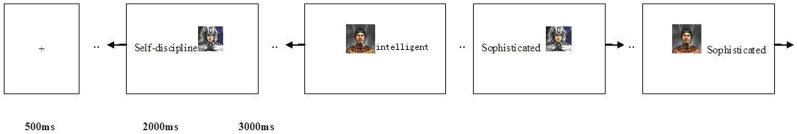
Positive avoidance – negative approach (inconsistent pattern, visual pictures). The Group 4 condition tested an inconsistent pattern based on visual pictures. A picture of the reference object appeared on the screen, and a corresponding personality adjective appeared on either the left or the right side of the picture.

**Figure 5:**
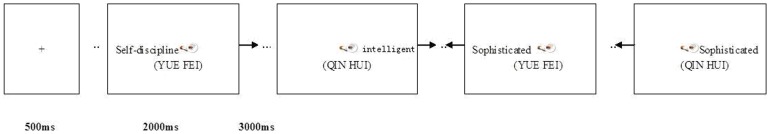
Positive approach - negative avoidance (consistent pattern, audition). The Group 5 condition tested a consistent pattern based on auditory stimuli. The pronunciation of the name of the reference object appeared in the center of the screen, and a corresponding personality adjective appeared at either the left or the right of this pronunciation.

**Figure 6:**
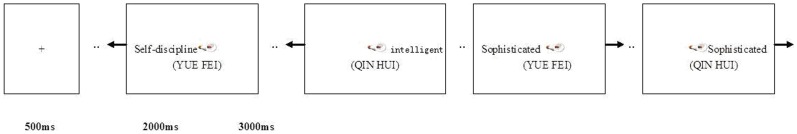
Positive avoidance – negative approach (inconsistent pattern, audition). The Group 6 condition tested an inconsistent pattern based on audition. The pronunciation of the name of a reference object was displayed in the center of the screen, and a corresponding personality adjective appeared at either the left or the right side of the pronunciation.

Experimental flow diagrams are presented in Figs. 1 to 6. All of the experimental materials were presented through the DMDX stimulus presentation system, which includes 6 subsystems with the same test frequency of 32. In the experiment, when a tip-stimulated black ‘+’ appeared within 500 ms, the subjects were instructed to focus on the screen. The test stimulus appeared at 2000 ms. The participants were asked to respond by pressing the arrow key within 3000 ms. After showing an empty screen for an additional 1000 ms, the system ran the next experimental item. The Group 1 condition tested a consistent pattern based on visual words. A reference name appeared on the center of the screen, and a corresponding personality adjective appeared on either the left or the right side of the reference name. To distinguish between the two levels of valence of stimuli, the study set up personality adjectives (positive or negative), reference objects, and movement adjectives to determine consistent effect reaction times for the different directions of movement. When YUE FEI and a commendatory word appeared, the subjects were asked to click the arrow (starting from the personality adjective) pointed toward YUE FEI (for instance, in ‘YUE FEI Intelligent’, the instruction was to press the ‘←’ key). Approach or avoidance action was reflected in the ‘←’ key or ‘→’ key responses. The computer automatically recorded the reaction time. When the screen presented YUE FEI and a derogatory word, the subjects were asked to click the arrow directed away from YUE FEI. When the screen presented QIN HUI and a commendatory word, the subjects were expected to click the arrow (from the personality adjective) pointed away from QIN HUI. When QIN HUI and a derogatory word were presented, the subjects were asked to click the arrow (from the personality adjective) pointed toward QIN HUI. A practice module was used prior to the formal experiment. After the first group experiment, subjects were asked to take a two-minute break. The Group 2 condition tested an inconsistent pattern based on visual words. When the screen presented a combination of YUE FEI and a commendatory word, the subjects were asked to click the arrow directed away from YUE FEI (for example, in ‘YUE FEI intelligent’, the instruction was to press the ‘→’ key). When YUE FEI and a derogatory word appeared, the subjects were expected to click the arrow pointed toward YUE FEI. When QIN HUI and a commendatory word appeared, subjects were asked to click the arrow pointed toward QIN HUI. When QIN HUI and a derogatory word were presented, the subjects were asked to click the arrow directed away from QIN HUI. The Group 3 condition tested a consistent pattern based on visual pictures. A picture of the reference object was displayed on the screen, and a corresponding personality adjective appeared on either the left or the right side of the picture. Reaction model is similar to group 1. The Group 4 condition tested an inconsistent pattern based on visual pictures. A picture of the reference object appeared on the screen, and a corresponding personality adjective appeared on either the left or the right side of the picture. Reaction model is similar to group 2. The Group 5 condition tested a consistent pattern based on auditory stimuli. The pronunciation of the name of the reference object appeared in the center of the screen, and a corresponding personality adjective appeared at either the left or the right of this pronunciation. Reaction model is similar to group 1. The Group 6 condition tested an inconsistent pattern based on audition. The pronunciation of the name of a reference object was displayed in the center of the screen, and a corresponding personality adjective appeared at either the left or the right side of the pronunciation. Reaction model is similar to group 2. To eliminate the order effect, all experimental conditions were conducted randomly.

## Results

Incorrect responses (3.9%) and responses with latencies below 300 ms and above 4000 ms (0.8% of correct responses) were discarded (see Table 1 for RT data). The results were analyzed using a mixed ANOVA.

**Table 1:** RT of presentation mode.

	Gender	Mean	SD	N
Visual words-YUE FEI-positive-approach	Male	1415.43	263.49	12
	Female	1523.72	348.97	11
Visual words -YUE FEI- positive- avoidance	Male	1544.29	312.65	12
	Female	1644.75	199.99	11
YUE FEI- negative- avoidance	Male	1433.77	210.83	12
	Female	1728.87	395.50	11
Visual words -YUE FEI- negative-approach	Male	1660.76	514.79	12
	Female	1709.72	215.66	11
Visual words -QIN HUI-positive-approach	Male	1607.97	468.97	12
	Female	1549.93	234.47	11
Visual words -QIN HUI-positive-avoidance	Male	1444.41	201.92	12
	Female	1556.53	332.96	11
Visual words -QIN HUI-negative-approach	Male	1586.58	346.13	12
	Female	1661.55	343.79	11
Visual words -QIN HUI-negative-avoidance	Male	1615.26	403.71	12
	Female	1592.35	181.64	11
Visual pictures -YUE FEI-positive-approach	Male	1105.12	98.64	12
	Female	1326.63	209.25	11
Visual pictures -YUE FEI-positive- avoidance	Male	1496.83	245.00	12
	Female	1473.85	287.44	11
Visual pictures -YUE FEI- negative-avoidance	Male	1329.30	274.61	12
	Female	1460.81	328.08	11
Visual pictures -YUE FEI- negative-approach	Male	1424.53	265.44	12
	Female	1517.42	264.09	11
Visual pictures -QIN HUI-positive-approach	Male	1338.33	186.89	12
	Female	1497.11	350.26	11
Visual pictures -QIN HUI-positive-avoidance	Male	1272.08	257.98	12
	Female	1373.52	257.69	11
Visual pictures -QIN HUI-negative-approach	Male	1272.93	172.61	12
	Female	1522.03	340.77	11
Visual pictures -QIN HUI-negative-avoidance	Male	1358.79	199.75	12
	Female	1512.37	357.34	11
Auditory stimuli -YUE FEI-positive-approach	Male	3717.11	129.41	12
	Female	3712.02	202.40	11
Auditory stimuli -YUE FEI-positive- avoidance	Male	3810.54	159.04	12
	Female	3649.19	633.03	11
Auditory stimuli -YUE FEI- negative-avoidance	Male	3687.58	179.64	12
	Female	3752.37	234.60	11
Auditory stimuli -YUE FEI- negative-approach	Male	3605.61	141.01	12
	Female	3485.77	545.84	11
Auditory stimuli -QIN HUI-positive-approach	Male	3868.15	158.36	12
	Female	3722.35	661.36	11
Auditory stimuli -QIN HUI-positive-avoidance	Male	3800.51	122.55	12
	Female	3806.44	147.74	11
Auditory stimuli -QIN HUI-negative-approach	Male	3675.41	166.93	12
	Female	3753.72	175.49	11
Auditory stimuli -QIN HUI-negative-avoidance	Male	3714.82	143.83	12
	Female	3530.25	678.08	11

Incorrect responses and responses with latencies below 300 ms and above 4000 ms were discarded. Table 1 is Mean and SD of different experimental treatment.

The result shows that the presentation mode (*F* (2, 42) = 925.32, *p*<.001, Partial η^2^ = .98) has a significant effect on RT. The results show that the interaction between the presentation mode and the reference object (*F* (2, 42) = 3.42, *p*<.05, Partial η^2^ = .14) also has significant effects on RT. The interactions among the presentation mode, the reference object, and gender (*F* (2, 42) = 5.54, *p*<.01, Partial η^2^ = .21) have significant effects on RT as well. The same holds true for the interaction between the presentation mode and the valence of stimulus (*F* (2, 42) = 26.07, *p*<.001, Partial η^2^ = .55) and the interaction between the presentation mode and the direction of the arrow (*F* (2, 42) = 4.11, *p*<.05, Partial η^2^ = .16). Post-hoc analyses indicated significant differences in the levels of the presentation mode (RT _audition_>RT _visual words_>RT_ visual pictures_). In the analysis of simple effects, presentation mode had a significant impact on YUE FEI (*p*<.001), the positive valence (*p*<.001), and the approach direction (*p*<.001). A t-test based on visual pictures showed that the average compatibility effect was significantly different from zero, *t* (22) = 2.43, *p*<.05 (M = 485 ms). Based on visual pictures, the RT of the approach behavioral tendency (M = 1211 ms) was faster than the avoidance behavioral tendency (M = 1432 ms) when YUE FEI matched a positive word or QIN HUI matched a negative word.

## Discussion

### From visual research to auditory research

Perceptions of emotional content depend on auditory and visual cues [29]–[30], [32]. Visual stimuli have an affective stimulus–response (S–R) compatibility effect. The present study examined whether auditory stimuli has the same affective stimulus–response (S–R) compatibility effect as visual stimuli. The results suggest that the presentation mode has a significant effect on RT. The personality characteristics of the participants were considered in the study. As YUE FEI is a positive representation of character, individuals usually paid attention to their own good personality characteristics. Thus, the volunteers participated in the cognitive aspect of the YUE FEI evaluation. Cognitive processing was more reflected in the three combinations of a good personality that included YUE FEI, participants, and the experimental material. In evaluating QIN HUI, the cognitive processing of the participants was shortened. As long as information relating to QIN HUI was available, the individual possessed a certain mindset that QIN HUI and positive information could not be associated. Zhang proposed a meaning-spelling theory of Chinese characters, and stressed that Chinese characters made full use of the human brain's visual processing power. Compared to the alphabet, Chinese characters form more thorough, visual words. Chinese characters are considered more than mere tools for recording [33]. The analysis of visual pictures is reflective of a strong performance in visual processing power. Thus, the RT of visual pictures in the present study was the fastest. The results show that the presentation mode has a significant effect on RT, thereby necessitating auditory research. Significant differences exist in the levels of the presentation mode (RT _audition_>RT _visual words_>RT_ visual pictures_). Auditory stimuli add more to the cognition composition and are a type of meaning and serial processing. For auditory stimuli, the perception process of participants is successively. The study of spatial S–R compatibility of visual and auditory signals found that responses to visual signals were faster than those to auditory signal [34]. Their research further confirmed this point of view [35]. In some ways, these studies have consistency. In the visual experiment, the RT for word processing was slower than that for picture processing. This study agrees with the results of Zhang on one aspect, as their experimental material was also visual pictures [3], and indirectly supports the meaning-spelling theory of Chinese Characters [33].

The average compatibility effect was calculated, and a t-test based on visual pictures showed that it was significantly different from zero. However, the t-test also showed no significant difference for visual words (298 ms) or auditory stimuli (−243 ms). The compatibility effect value of auditory stimuli was the smallest. A smaller compatibility effect existed when switching the presentation mode from visual stimuli to auditory stimuli. This study found an interesting phenomenon in that the only significant compatibility effect for visual pictures was for Chinese. There are differences between the results of the present study and those of previous studies; therefore, more research is necessary to further validate these results.

In a previous study, the target and the referent matched (Einstein and positive words, Hitler and negative words), approach was faster avoidance [3]. This study found that the approach behavioral tendency was faster than the avoidance behavioral tendency when a positive character matched a positive adjective or a negative character matched a negative adjective. The study was also confined to the research category of visual pictures. More research in this area is also necessary for further validation.

### Research limitations and the prospect

As a keyboard was used to input experimental reaction in the study, some limitations may be present. Further study can use a real approach and avoidance action to conduct related research (such as placing circuit boards at the feet, when participants perform approach and avoidance actions; the connection of the reaction device to the circuit board collects relevant data and reaction type). The reference valence effects in the study of affective S–R compatibility by Augmented Reality (AR) technology can improve the ecological validity of the study (the immersive feeling from registered, calibration, tracking, and fusion technologies allow participants to pay more attention to the real psychological reaction).
